# In Vitro Evaluation of Probiotic Properties of Two Novel Probiotic Mixtures, Consti-Biome and Sensi-Biome

**DOI:** 10.4014/jmb.2303.03011

**Published:** 2023-06-19

**Authors:** You Jin Jang, Bonggyu Min, Jong Hyun Lim, Byung-Yong Kim

**Affiliations:** R&D Center, Chong Kun Dang Healthcare, Seoul 07249, Republic of Korea

**Keywords:** Probiotics, pathogens, antimicrobial activity, immune response, short-chain fatty acid, intestinal disorder

## Abstract

Changes in the gut microbiome cause recolonization by pathogens and inflammatory responses, leading to the development of intestinal disorders. Probiotics administration has been proposed for many years to reverse the intestinal dysbiosis and to enhance intestinal health. This study aimed to evaluate the inhibitory effects of two newly designed probiotic mixtures, Consti-Biome and Sensi-Biome, on two enteric pathogens *Staphylococcus aureus* and *Escherichia coli* that may cause intestinal disorders. Additionally, the study was designed to evaluate whether Consti-Biome and Sensi-Biome could modulate the immune response, produce short-chain fatty acids (SCFAs), and reduce gas production. Consti-Biome and Sensi-Biome showed superior adhesion ratios to HT-29 cells and competitively suppressed pathogen adhesion. Moreover, the probiotic mixtures decreased the levels of pro-inflammatory cytokines, such as tumor necrosis factor-α, interleukin (IL)-6 and IL-1β. Cell-free supernatants (CFSs) were used to investigate the inhibitory effects of metabolites on growth and biofilms of pathogens. Consti-Biome and Sensi-Biome CFSs exhibited antimicrobial and anti-biofilm activity, where microscopic analysis confirmed an increase in the number of dead cells and the structural disruption of pathogens. Gas chromatographic analysis of the CFSs revealed their ability to produce SCFAs, including acetic, propionic, and butyric acid. SCFA secretion by probiotics may demonstrate their potential activities against pathogens and gut inflammation. In terms of intestinal symptoms regarding abdominal bloating and discomfort, Consti-Biome and Sensi-Biome also inhibited gas production. Thus, these two probiotic mixtures have great potential to be developed as dietary supplements to alleviate the intestinal disorders.

## Introduction

Probiotics are live microorganisms with proven health benefits, when administrated in adequate amounts to the host [1]. The use of probiotics has been commonly recommended for the safe and effective management of intestinal disorders such as constipation and diarrhea in which normal microbiome is disrupted by infectious pathogens, diet or antibiotics [2, 3]. Protective roles of probiotics against pathogens and the relieving mechanisms of intestinal disorders have received considerable attention. Pathogen inhibition by probiotics might protect the host from infection as a natural barrier against exposure in the gastrointestinal tract [4]. In particular, probiotics aid in suppressing pathogen attachment to the intestinal epithelium, producing chemical defenses, and reducing the gas produced in the gut [4-7]. They also regulate pro-inflammatory molecules induced by pathogen infection of the intestinal epithelium [5, 8]. Various pro-inflammatory cytokines, including tumor necrosis factor-α (TNF-α), interleukin (IL)-6, and IL-1β are involved in intestinal inflammation [6]. Key mediators of the interaction between probiotics and gut health are microbial metabolites, particularly short-chain fatty acids (SCFAs) [9]. SCFAs, primarily acetic, propionic, and butyric acids, produced by intestinal bacteria through fermentation, prevent pathogen attachment through colonization and exhibit potent antimicrobial and anti-inflammatory functions [10].

Although majority of the studies generally focus on a single strain, there is increasing interest in the potential effects of probiotic mixtures and evidences of their synergistic effects compared to the effects of single strains [7, 11, 12]. An in vitro study has demonstrated that probiotic mixtures can inhibit enteric pathogens more efficiently than their single strain preparation [12]. In a previous study, probiotics mixture, namely LACTO 5X, containing several species of bacteria, could alleviate loperamide-induced constipation and improve intestinal microbiota in animal experiments [13]. Several strains composed of the probiotic mixtures used in our study have been shown to be effective in clinical trials for inflammatory bowel syndrome. For example, administration of the strains SynBalance SmilinGut (*Lactiplantibacillus plantarum* PBS067, *Lacticaseibacillus rhamnosus* LRH020, and *Bifidobacterium animalis* ssp. *lactis* BL050), contained in Consti-Biome alleviated constipation-related symptoms [14] and Sensi-Biome (*B. lactis* UABla-12 and *Lactobacillus acidophilus* DDS-1) relieved diarrhea-related symptoms [15].

In this study, we evaluated two probiotic mixtures with in vitro experiments for potential pathogen inhibiting and anti-inflammatory properties, and further demonstrated the evidence supporting effects on intestinal disorders. We selected two pathogens, *Staphylococcus aureus* and *Escherichia coli* which cause various gastrointestinal infections. Probiotics were tested to inhibit the pathogens and treat intestinal disorders [3]. According to the association between the two pathogens and the intestinal microbiota of people with intestinal disorders, *S. aureus* is abundant in the feces of patients with chronic constipation [16]. Also, *S. aureus* infection is significantly involved in the functional pathways of differentially expressed genes in slow transit constipation [17]. In contrast, *E. coli* known to produce toxins leading to diarrhea, is significantly more abundant in the feces of patients with diarrhea than healthy subjects [18, 19].

The aim of this study was to evaluate two probiotic mixtures, Consti-Biome and Sensi-Biome, for their potential application as effective dietary supplements to alleviate intestinal disorders. We demonstrated the intestinal health-promoting properties of two probiotic mixtures, notably inhibitory effects on pathogens which may cause intestinal disorders, immunomodulation, and the production of SCFAs.

## Materials and Methods

### Preparation of Pathogenic Bacteria and Probiotic Mixtures

The pathogenic bacteria, *S. aureus* ATCC 6538 and *E. coli* ATCC 8739 were cultured in tryptic soy broth (TSB; MB Cell, Korea) and Luria-Bertani broth (LB; MB Cell), respectively. The strains included in the probiotic mixtures, Consti-Biome and Sensi-Biome, are listed in Table 1. Consti-Biome consisted of six probiotic strains including SynBalance SmilinGut (*B. lactis*, *L. plantarum*, and *L. rhamnosus*; Roelmi HPC, Italy), *L. plantarum* (Chr. Hansen, Denmark), *L. acidophilus* (Chr. Hansen), and *S. thermophilus* (Chong Kun Dang Bio, Korea). Sensi-Biome also consisted of six probiotic species, including *B. lactis* (Chr. Hansen), *B. bifidum* (Danisco, Denmark), *L. acidophilus* (Chr. Hansen), *L. plantarum* (Chr. Hansen), *S. thermophilus* (Chong Kun Dang Bio) and *Lactococcus lactis* (*Lc. lactis*; Mediogen, Korea). The two probiotic mixtures were adjusted to 10^10^ colony-forming units (CFU) and diluted to the concentration required for the experiment using Dulbeccós phosphate-buffered saline (D-PBS; Welgene, Korea). Bacteria were cultured in deMan-Rogosa-Sharpe (MRS) broth (BD Difco, USA) and sub-cultured twice before use.

### Cell Culture

HT-29 cell line was used for intestinal adhesion of probiotic mixtures and competitive exclusion of pathogens, and RAW264.7 cell line was used for immune response experiments. The human colorectal adenocarcinoma cell line, HT-29, was cultured in Roswell Park Memorial Institute (RPMI)-1640 medium (Thermo Fisher Scientific, USA) supplemented with 10% (v/v) heat-inactivated fetal bovine serum (FBS; Gibco, USA) and 100 U/ml penicillin-streptomycin (Gibco). The mouse macrophage cell line, RAW264.7, was maintained in Dulbecco’s modified Eagle medium (Gibco) supplemented with 10% (v/v) FBS and 100 U/ml penicillin-streptomycin. Cells were incubated at 37°C in a 5% CO_2_ incubator and the medium was replaced every 2–3 days.

### Bacterial Adhesion Assay with HT-29 Cell Line

The adhesion abilities of the probiotic mixtures, Consti-Biome and Sensi-Biome, to the intestinal cell line, HT-29, were determined. HT-29 was cultured in RPMI-1640 medium supplemented with 10% FBS on the six-well cell plates and incubated at 37°C in a 5% CO_2_-containing atmosphere. After forming a conﬂuent monolayer, the cells were washed twice with D-PBS (pH 7.3). Consti-Biome and Sensi-Biome cultured in 10 ml MRS broth were harvested and washed thrice with D-PBS. Bacterial pellets were resuspended in RPMI-1640 medium at 10^8^ CFUs. Monolayers of HT-29 cells grown in six-well cell plates were inoculated with 2 ml fresh culture medium and 100 μl of bacterial suspensions and incubated at 37°C under 5% CO_2_ for 2 h. After incubation, each well was washed thrice with D-PBS, to remove non-adherent bacteria, and digested using a lysis solution (0.25% trypsin-EDTA). Serial dilutions of the adherent bacteria were plated on MRS agar and incubated at 37°C for 24 h and the number of bacteria was measured as following equation:

Adhesion Ratio (%) = [viable cells (log CFU/ml) / initial cells (log CFU/ml)] × 100

Bacterial adherence to HT-29 cells was visualized after ﬁxation using methanol (Sigma-Aldrich, USA) and staining using Giemsa (Sigma-Aldrich). Gram staining was used to visualize gram-positive lactic acid bacteria (LAB) adhering to the cells. Images were obtained using light microscopy with a 100× oil immersion objective.

### Competitive Exclusion Assay

A competitive exclusion assay was performed, as described by Wang *et al* [20] with minor modifications. Approximately 5 × 10^5^ HT-29 cells per well were cultivated in six-well plates to 80–90% confluence and used for adhesion experiments. Bacterial cells (*S. aureus* and *E. coli*) were washed twice with D-PBS, and 100 μl of *S. aureus* or *E. coli* suspension (10^8^ CFUs) and 100 μl of Consti-Biome or Sensi-Biome suspension (10^8^ CFUs) were added to each well simultaneously. The cultures were incubated at 37°C for 90 min; the monolayers were then washed twice with D-PBS and digested using trypsin (0.25%). Serial dilutions of the adherent bacteria were plated on Baird-Parker agar (MB Cell) with egg yolk tellurite emulsion (MB Cell) for *S. aureus* and MacConkey Agar (MB Cell) for *E. coli* and incubated at 37°C for 24 h. The number of colonies was then counted.

### Cell Viability Assay

The number of viable cells was determined by the mitochondrial ability to convert 3-(4, 5-dimethylthiazol-2-yl)-2, 5-diphenyltetrazolium bromide (MTT) to formazan. The effects of the Consti-Biome and Sensi-Biome on cell viability of RAW264.7 cells were evaluated using the MTT (Sigma-Aldrich) assay. RAW264.7 cells (1 × 10^5^ cells/ml) were plated in 96-well cell plates (SPL Life Sciences, Korea) and incubated for 24 h at 37°C in a 5% CO_2_ incubator. Consti-Biome and Sensi-Biome were then treated at 1 × 10^5^, 5 × 10^5^, 1 × 10^6^, 5 × 10^6^, 1 × 107, 5 × 107, and 1 × 10^8^ CFU/ml for 24 h at 37°C. After aspiration of the supernatant, the cells were treated with the MTT solution (2.5 mg/ml in D-PBS) and incubated for 4 h. After discarding the supernatant, dimethyl sulfoxide (Sigma-Aldrich) was added to each well and the generated formazan deposits were dissolved. The absorbance of each well was measured at 570 nm using a microplate reader [21]. Cell viability was calculated as the percentage absorbance compared to that of untreated cells, which served as a control, using the following equation:

Cell viability (%) = [OD_(sample)_/OD_(control)_] × 100

### Measurement of Cytokine Levels

To investigate the effect of Consti-Biome and Sensi-Biome on cytokine levels in lipopolysaccharides (LPS)-treated cells, RAW264.7 cells (3 × 10^5^ cells) seeded into 24-well plates (SPL Life Sciences) were pretreated with Consti-Biome and Sensi-Biome (10^7^ CFU/ml) for 2 h prior to treatment with 1 μg/ml LPS (*E. coli* O111:B4, Sigma-Aldrich) for 24 h at 37°C in a 5% CO_2_ incubator. Cell-free supernatants (CFSs) were collected from each well and stored at -20°C until assayed for cytokine levels. The concentrations of TNF-α, IL-6, and IL-1β in the supernatants of RAW264.7 cell cultures were determined using an enzyme-linked immunosorbent assay (ELISA) kit, according to the manufacturer’s instructions (BioLegend, USA). Cytokine concentrations in the test samples were evaluated with reference to standard curves.

### Antimicrobial Activity of CFS

CFSs were prepared to validate the ability of Consti-Biome and Sensi-Biome to antagonize pathogens [22]. After culturing in MRS, CFSs were obtained from the cultured bacteria using centrifugation at 4,000 ×*g* for 20 min. The cell pellets were discarded and the supernatants were filtered through a 0.2 μm filter. *S. aureus* and *E. coli* cells at a density of 10^6^ CFU/ml were seeded into the wells of a 96-well microplate (SPL Life Sciences), and 0, 5, 10, 20, or 40% (v/v) CFSs of Consti-Biome or Sensi-Biome were added to each well. The absorbances of *S. aureus* and *E. coli* at an optical density (OD) of 600 nm were monitored at 10 h [20]. The inhibition ratio of bacterial growth was calculated as a percentage of absorbance compared with untreated cells, which served as a control, using the following equation:

Inhibition Ratio (%) = [1 − OD_(sample)_/OD_(control)_] × 100

### Biofilm Inhibitory Assay

The effect of CFS on the bioﬁlm inhibition of both *S. aureus* and *E. coli* was determined using crystal violet (CV) staining, as described previously [23]. Suspensions of the two pathogens (10^8^ CFU/ml each) were cultured in 12-well cell culture plates containing sterile TSB and LB. The CFSs of Consti-Biome and Sensi-Biome were diluted to 20% and 40%, respectively, and added to each well. In the control wells, the CFSs were replaced with sterile MRS broth. After incubation at 37°C for 10 h, non-adherent cells were removed by washing the well plates twice using D-PBS. Subsequently, a 0.1% CV solution was added to the wells and incubated for 10 min. The excess CV was then removed by washing with D-PBS. Following this, the ﬁxed CV was released by washing with 30% acetic acid. Finally, the OD of the biofilm-related CV was measured at a wavelength of 570 nm. The experiment was conducted in triplicates. The inhibition ratio of the biofilm was calculated as a percentage of absorbance compared with that of untreated cells, which served as a control, using the same equation as for antimicrobial activity.

### Fluorescence Microscopy and Scanning Electron Microscopy Analysis

To visualize the antimicrobial activity of CFSs, *S. aureus* and *E. coli* cells were stained using the LIVE/DEAD BacLight Bacterial Viability Kit (Life Technologies, USA), based on the manufacturer’s instruction. After culturing the *S. aureus* and *E. coli* strains, the cell pellets were resuspended in 40% CFSs of Consti-Biome and Sensi-Biome, respectively, and incubated at 37°C for 10 h. In the control tube, the CFSs were replaced with sterile MRS broth. Dual fluorescent stain (20 μl) was added to 1 ml of cell suspension containing CFS-treated and control cells, and incubated in the dark at room temperature (25°C) for 20 min. The remaining dye was removed by discarding the supernatant after centrifugation at 10,000 × *g* for 5 min. The obtained cell pellets were resuspended in 0.85% NaCl solution. Fluorescence microscopy (Leica, Germany) was performed under dark conditions within 1 h, to avoid reducing fluorescence intensity.

For scanning electron microscopy (SEM) analysis to observe the pathogen structures, 10^8^ CFU/ml inoculums of *S. aureus* and *E. coli* were treated with 40% CFS of Consti-Biome and Sensi-Biome, respectively, and incubated at 37°C for 10 h. Non-treated *S. aureus* and *E. coli* culture served as controls. After washing with fresh D-PBS, the cells were fixed with 2.5% glutaraldehyde for 12 h. The bacteria were then washed thrice with demineralized water for 5 min each. After dehydration with ethanol (stepwise gradients of 30, 50, 70, 80, 90, and 100%) for 15 min, the specimens were dried with hexamethyldisilazane and coated with gold. Cell morphology was examined and imaged using field-emission SEM.

### Analysis of Short-Chain Fatty Acids Using Gas Chromatography

To determine the production of short-chain fatty acids (SCFAs) such as acetic, propionic, and butyric acid, suspensions of the nine single strains and two probiotic mixtures, Consti-Biome and Sensi-Biome were adjusted 10^8^ CFU/ml each and were cultured on MRS broth at 37°C for 24 h. These were centrifuged at 4,000 ×*g* for 20 min and the supernatants were filtered using 0.2 μm membrane filters. SCFAs were identified based on their specific retention times using an Agilent 8890 gas chromatograph (GC) system (Agilent Technologies, USA) equipped with a flame ionization detector (FID). The capillary chromatographic column used was a nitroterephthalic acid modified polyethyleneglycol column (DB-FFAP, 50 m, 0.32 mm i.d., 0.50 μm film thickness, purchased from Agilent Technologies). The GC injector was maintained at 250°C and injection was performed in the split mode (split ratio, 50:1). The oven temperature was initially set at 50°C for 1 min, programmed at a rate of 25°C/min to 200°C, and then at 4°C/min to 230°C, resulting in a total run time of 14.5 min. The FID temperature was maintained at 280°C. The flow rates of hydrogen and air (used as the makeup gases) were 40 and 450 ml/min, respectively. Nitrogen was used as the carrier gas at a flow rate of 30 ml/min. The injected sample volume for the GC analysis was 1 μl. Standards for volatile SCFAs were obtained from Sigma-Aldrich. To quantify the peak area in terms of concentration, a calibration curve of SCFAs ranging from 5 to 500 μg/ml was drawn using the Agilent ChemStation software.

### Evaluation of Gas Production Inhibition

The ability of Consti-Biome and Sensi-Biome to inhibit gas production by *E. coli* was evaluated, as described by Monteiro *et al* [24]. The assay was performed by inoculating 30 μl (10^8^ CFU/ml) of *E. coli* culture into the upper one-third layer of the LB agar (supplemented with 1.5% bacteriological agar; 3 ml per tube). Subsequently, 3 ml of MRS broth containing 0.7% bacteriological agar was melted, cooled to 50°C, and inoculated with 30 μl (10^8^ CFU/ml) of Consti-Biome and Sensi-Biome cultures. The contents were homogenized by vortexing and immediately poured over the LB agar layer into tubes inoculated with *E. coli* strain. LB agar with *E. coli* and MRS agar without the inoculated probiotics were used as negative controls. The tubes were incubated under aerobic conditions at 37°C for 24 h.

### Statistical Analyses

All experimental results are expressed as mean ± standard deviation (SD) of independent experiments performed in triplicates. Analyses were performed using Student's t-test and visualized using GraphPad Prism 5.01 (GraphPad Software, USA). *p*-values < 0.05 were considered statistically significant.

## Results

### Adhesion Ability of Consti-Biome and Sensi-Biome to HT-29 Cells

**Fig. 1:**
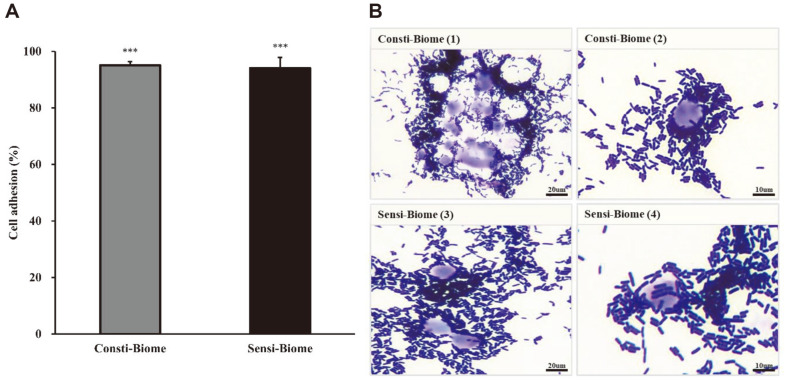
Cell adhesion activity of Consti-Biome and Sensi-Biome to HT-29 cells. (**A**) Adherence ability of Consti- Biome and Sensi-Biome to HT-29 cells. Bar charts show the mean ± standard deviation of three independent experiments. A significant difference compared with that of the untreated strains was indicated as, ***p < 0.001 (**B**) Microscopic images of adhesion assay. Consti-Biome (1, 2) and Sensi-Biome (3, 4) adhered to HT-29 cells were stained by Giemsa- and Gram-staining assays and examined by light microscopy under a 100× oil immersion objective.

The ability to adhere to intestinal epithelial cells and colonization are important criteria for the selection of probiotics which can be established in the intestine [25]. The effects of Consti-Biome and Sensi-Biome on the initial adherence to the intestinal cell line HT-29 was evaluated by plating and light microscopy. Adhesion ability was calculated as a percentage of adherence values. The adherence ratio of the Consti-Biome and Sensi-Biome groups were 95.05 ± 1.34% and 94.03 ± 3.81%, respectively (Fig. 1A). The adhesion efficiency was further validated by visualizing adherent bacteria using Giemsa and Gram staining. Microscopic images showed an overall adhesion capability of both stained Consti-Biome and Sensi-Biome to HT-29 cells (Fig. 1B).

### Anti-Adhesion Effects of Consti-Biome and Sensi-Biome against Pathogens

**Fig. 2:**
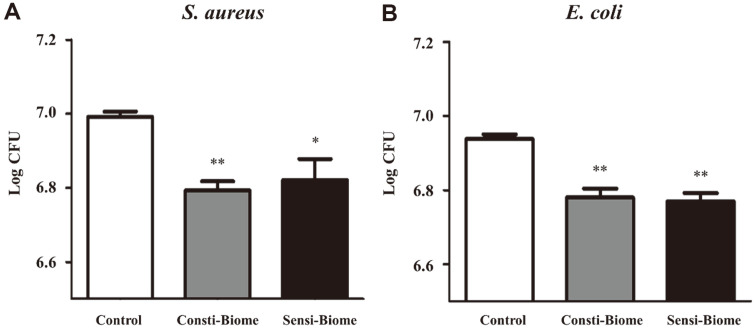
Inhibitory effect of Consti-Biome and Sensi-Biome on the adhesion of pathogens to HT-29 cell. HT-29 cells were incubated with (**A**) *S. aureus* and (**B**) *E. coli* alone (Control) or co-incubation with 100 μl of Consti-Biome and Sensi- Biome (10^8^ colony forming unit (CFU)) for 90 min. Cell cultures of pathogens were plated on Baired-Parker agar with egg yolk tellurite emulsion for *S. aureus* and MacConkey Agar for *E. coli* to determine viable cell counts. The agar plates were incubated at 37°C for 24 h and the number of pathogens CFUs bound to HT-29 cells were estimated. The values are expressed as the mean ± standard deviation. A significant difference from the control was indicated as, *p < 0.05, or **p < 0.01.

Probiotics competitively inhibit pathogen binding, thereby hindering their colonization [26]. We investigated the anti-adhesion properties of these pathogens using a competition assay. In bacterial control wells, average 6.99 log CFU/ml (*S. aureus*) and 6.94 log CFU/ml (*E. coli*) were retained after D-PBS washes. In the test wells, the number of pathogen cells binding to HT-29 cells was significantly reduced when co-cultured with Consti-Biome or Sensi-Biome. The average *S. aureus* counts were 6.79 log CFU/ml in Consti-Biome- and 6.82 log CFU/ml in Sensi-Biome-treated cells (Fig. 2A). The average *E. coli* counts were 6.78 log CFU/ml in Consti-Biome- and 6.77 log CFU/ml in Sensi-Biome-treated cells (Fig. 2B). These results suggest that Consti-Biome and Sensi-Biome compete with pathogenic bacteria and prevent them from adhering to HT-29 cells.

### Modulation on LPS-Induced Pro-Inflammatory Cytokines

**Fig. 3:**
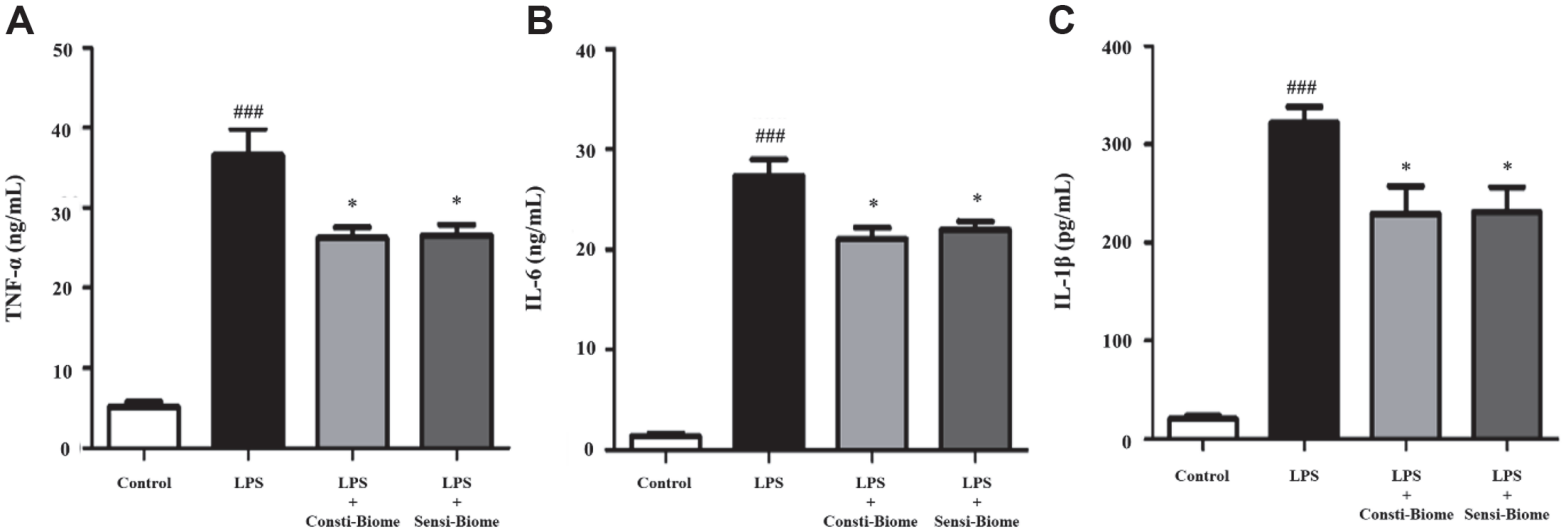
Effect of Consti-Biome and Sensi-Biome on pro-inflammatory cytokines in LPS-stimulated RAW264.7 cell. RAW264.7 cells were treated with Consti-Biome and Sensi-Biome (10^7^ CFU/ml) for 2 h followed by LPS (Lipopolysaccharides) stimulation (1 μg/ml). After incubation for 24 h, the supernatants were taken, and the levels of (**A**) Tumor necrosis factor (TNF)-α, (**B**) interleukin (IL)-6 and (**C**) IL-1β were measured by ELISA. The values are expressed as the mean ± standard deviation. ^###^*p* < 0.001 vs. control cells (white-colored bar). *p < 0.05 vs. LPS-treated cells (black-colored bar).

We examined the cytotoxic activity of different Consti-Biome and Sensi-Biome concentrations on RAW264.7 cells using the MTT assay. Treatment of RAW264.7 cells for 24 h with Consti-Biome and Sensi-Biome up to 1 × 10^7^ CFU/ml did not affect cell viability (Fig. S1). To determine the effect of probiotics on pro-inflammatory cytokines, RAW264.7 cells were stimulated with LPS, leading to effective macrophage activation [27] and then treated with Consti-Biome and Sensi-Biome. The concentrations of TNF-α, IL-6, and IL-1β in the culture supernatants of RAW 264.7 cells were measured using ELISA. LPS treatment of RAW 264.7 cells alone significantly increased cytokine production compared with the control. Compared to the LPS-stimulated cells, those treated with Consti-Biome and Sensi-Biome showed significantly decreased TNF-α, IL-6, and IL-1β levels (Fig. 3). Thus, Consti-Biome and Sensi-Biome may possess anti-inflammatory activities.

### Inhibitory Effect of CFSs on Pathogen Growth

**Fig. 4:**
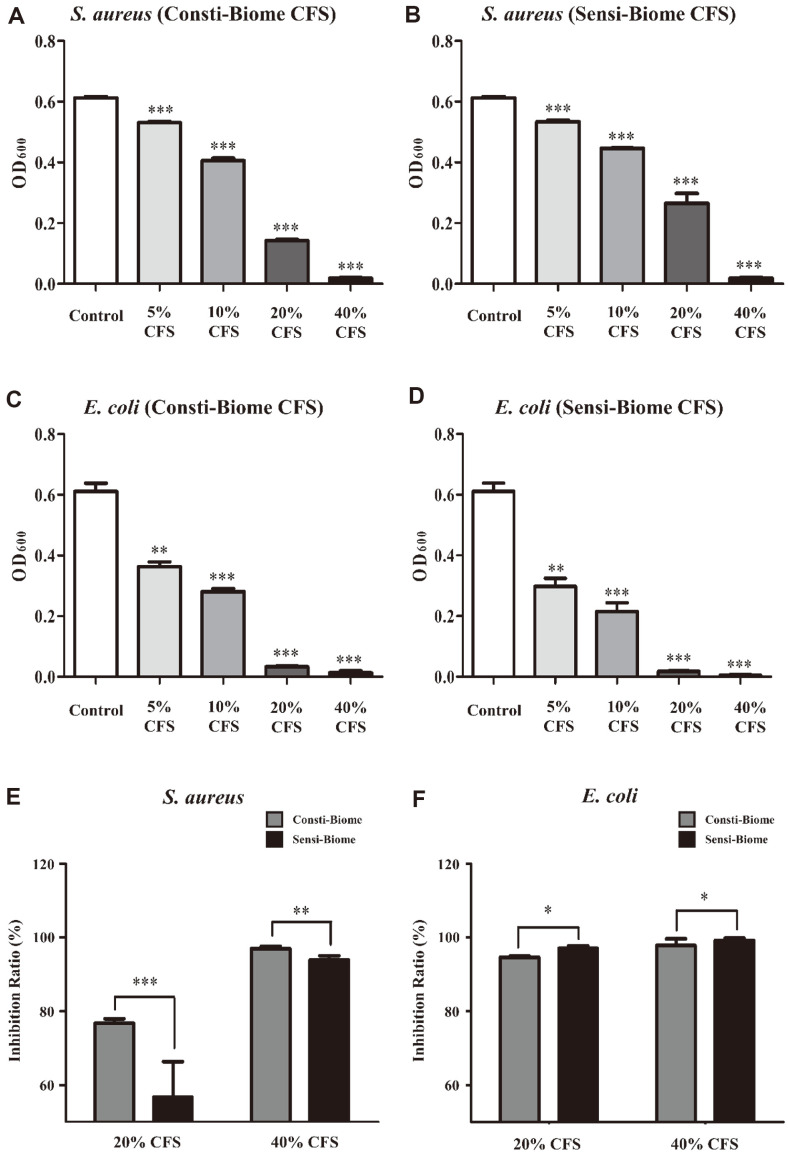
Antimicrobial activity of Consti-Biome and Sensi-Biome supernatants against pathogens. The inhibition of (**A, B**) *S. aureus* and (**C, D**) *E. coli* were observed in untreated (Control) or treated with four different concentrations (5, 10, 20, 40%) cell free supernatant (CFS) of Consti-Biome and Sensi-Biome using optical density (OD) at 600 nm. (**E, F**) The growth inhibition ratio of two pathogens was compared between CFSs of Consti-Biome and Sensi-Biome. The values are expressed as the mean ± standard deviation. A significant difference from the control was indicated as, *p < 0.05, **p < 0.01, or ***p < 0.001.

Antimicrobial activity is due to the production of metabolites such as organic acids, bacteriocins, and other compounds with inhibitory properties [28, 29]. To investigate antimicrobial activities, such as inhibition of growth and biofilm formation, sterile filtered CFSs containing metabolites from Consti-Biome and Sensi-Biome were prepared and added to *S. aureus* and *E. coli* cells at concentrations of 5, 10, 20, and 40%. The CFSs of Consti-Biome and Sensi-Biome were tested for concentration-dependent growth inhibitions of *S. aureus* and *E. coli*. All CFSs significantly reduced the growth of *S. aureus* and *E. coli* compared to the control not treated with CFS measured by the absorbance at OD600, indicating antimicrobial activity (Fig. 4A-4D). To identify which probiotics are more effective against pathogens, we compared their effects on *S. aureus* and *E. coli*. When comparing the two probiotic mixtures, Consti-Biome more effectively inhibited the growth of *S. aureus* than that with Sensi-Biome at CFS concentrations of 20% (76.77 ± 1.18% and 56.69 ± 9.66%, respectively) and 40% (96.95 ± 0.63% and 93.85 ± 1.13%, respectively) (Fig. 4E). In contrast, Sensi-Biome inhibited the *E. coli* growth more effectively than that with Consti-Biome at CFS concentrations of 20% (97.05 ± 0.71% and 94.6 ± 0.36%, respectively) and 40% (99.13 ± 0.66% and 97.82 ± 1.78%, respectively) (Fig. 4F).

### Effect of Anti-Biofilm Formation of CFSs against Pathogens

**Fig. 5:**
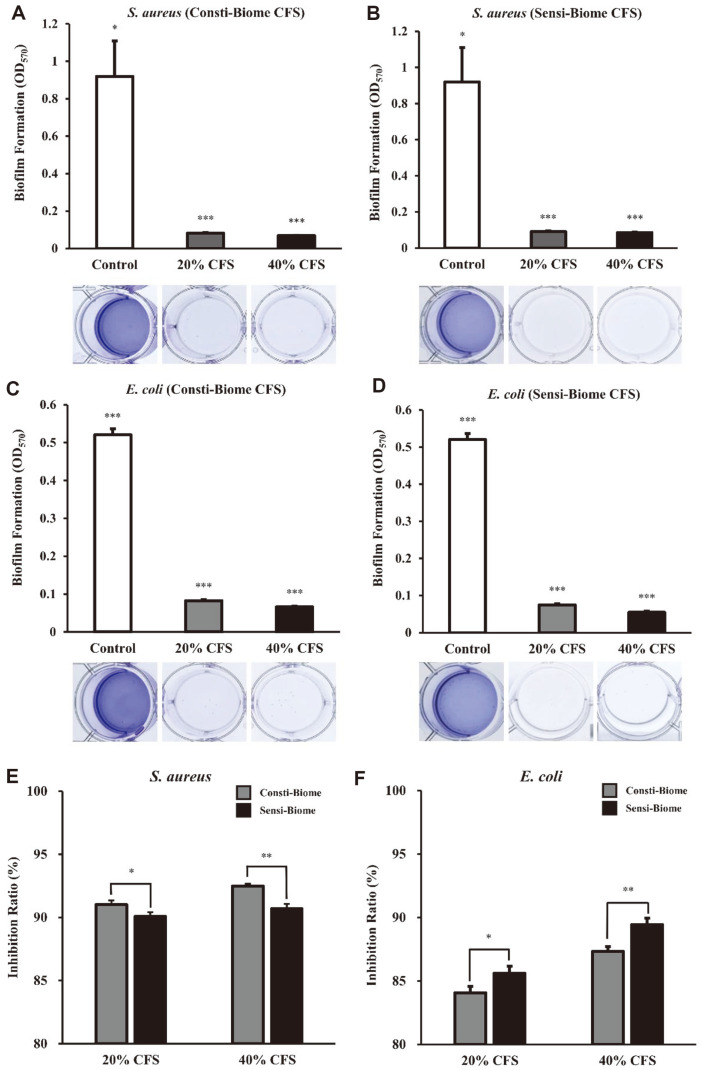
Anti-biofilm activity of Consti-Biome and Sensi-Biome supernatants against pathogens. Biofilm inhibitory by cell-free supernatants (CFS) of Consti-Biome and Sensi-Biome was evaluated by modified crystal violet assay performed in the 12-well cell culture plates. (**A, B**) *S. aureus*, (**C, D**) *E. coli*. Images of biofilm inhibition by CFS were shown below the graphs. The biofilm inhibition ratio of (**E**) *S. aureus* and (**F**) *E. coli*. Inhibition of biofilm formation were observed in untreated (Control) or treated with different concentrations (20 and 40%) CFS of Consti-Biome and Sensi-Biome. Bars are representative of the mean and error bars are representative of the standard deviation of three independent experiments. A significant difference from the control was indicated as, *p < 0.05, **p < 0.01, or ***p < 0.001.

Further, we evaluated the biofilm inhibitory activity of 20 and 40% CFSs from Consti-Biome and Sensi-Biome on *S. aureus* and *E. coli*. Biofilms are complex polymicrobial structures containing microorganisms and an extracellular matrix, the formation of which can resist extreme environmental conditions [23]. A concentration-dependent inhibition of biofilm formation was observed after treatment with Consti-Biome and Sensi-Biome CFSs (Fig. 5). Biofilms formed by *S. aureus* and *E. coli* were inhibited after exposure to Consti-Biome and Sensi-Biome CFSs. The biofilms of *S. aureus* and *E. coli* were more effectively inhibited by Consti-Biome and Sensi-Biome, respectively. The inhibition ratio of *S. aureus* biofilm formation was 91.01 ± 0.33% of Consti-Biome and 90.07 ± 0.35% of Sensi-Biome at 20% CFS, respectively. However, at a CFS concentration of 40%, Consti-Biome tended to inhibit biofilm formation more than effectively than that with Sensi-Biome (92.50 ± 0.19% and 90.69 ± 0.38%, respectively). In case of *E. coli*, the CFS of Sensi-Biome inhibited biofilm formation more effectively than that of Consti-Biome. At 20% CFS concentration, the biofilm inhibition ratios of Consti-Biome and Sensi-Biome were 84.07 ± 0.51% and 85.60 ± 0.58%, respectively. At 40% CFS concentration, the biofilm inhibition ratios of Consti-Biome and Sensi-Biome were 87.33 ± 0.38% and 89.44 ± 0.51%, respectively.

### Visualization of Antimicrobial Activity Using Fluorescence Microscopy and SEM

**Fig. 6:**
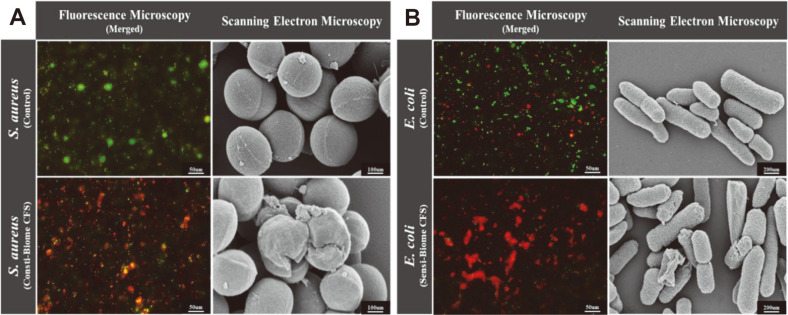
Fluorescence microscopy and scanning electron microscopy (SEM) images of *S. aureus* and *E. coli* in the presence of supernatants. (**A**) Microscopic images of *S. aureus* and (**B**) *E. coli* cells. Fluorescence microscopy images present fluorescent-stained *S. aureus* and *E. coli* cells after 10 h of cultivation containing 40% CFS of Consti-Biome and Sensi- Biome (Left in A and B). Cells were stained using the LIVE/DEAD Bacterial Viability kit. Live cells (SYTO-9, green) and dead cells (propidium iodide, red). Scale bar indicate 50 μm. SEM images present *S. aureus* and *E. coli* cells after 10 h of cultivation (Right in A and B). SEM images show structural damage of *S. aureus* cultivated in medium containing 40% CFS of Consti- Biome and *E. coli* in 40% CFS of Sensi-Biome. *S. aureus* images were observed in the scale of 100 nm with magnification of 100 KX and *E. coli* images were in the scale of 200 nm with magnification of 50 KX.

Microscopic evaluation further conﬁrmed the antimicrobial properties of the 40% CFSs through inhibition of both the pathogens. The inhibitory effects of the CFSs of Consti-Biome on *S. aureus* and Sensi-Biome on *E. coli* were examined microscopically using fluorescence staining assay and SEM (Fig. 6). To visualize the antimicrobial effects, *S. aureus* and *E. coli* were treated with 40% Consti-Biome and Sensi-Biome CFSs. Microscopic images showed a considerable reduction in the live bacterial count after 10 h of incubation with the CFSs. These changes were confirmed by the increasing number of dead bacteria (red) in the CFS-treated groups compared with those in the control group, as observed by LIVE/DEAD staining. Further, SEM was used to visualize the cell morphology of *S. aureus* and *E. coli* after treatment with 40% CFS. SEM observations revealed that, compared with the pathogen morphologies in the untreated control, Consti-Biome and Sensi-Biome CFSs destroyed the spherical shape of *S. aureus* and the rod-shaped structure of *E. coli* cells. These results indicate that Consti-Biome and Sensi-Biome CFSs may inhibit pathogen growth by damaging the cell wall. Thus, fluorescence microscopy and SEM images showed the antimicrobial activity of Consti-Biome and Sensi-Biome in influencing viability and altering morphological structures of the pathogens.

### Production of SCFAs

**Fig. 7:**
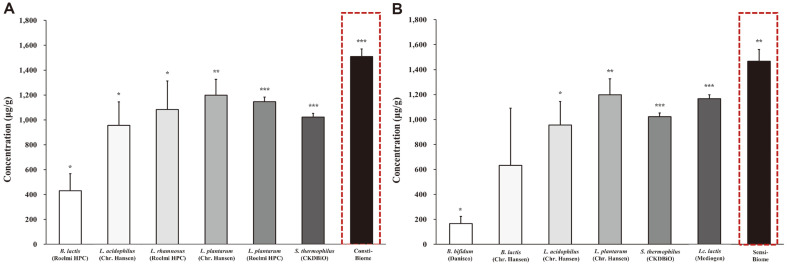
Comparison of the concentrations of total short-chain fatty acids (SCFAs) produced by single strains and two probiotic mixtures in supernatants. (**A**) Total SCFA concentrations produced by six single strains that make up the Consti-Biome and a probiotic mixture Consti-Biome, (**B**) six single strains that make up the Sensi-Biome and probiotic mixture Sensi-Biome in their supernatants, respectively. Bars are representative of the total SCFAs, which are the sum of acetic, propionic and butyric acids in the supernatant and the error bars are representative of standard deviation. The experiments were performed three times. A significant difference from the control was indicated as, *p < 0.05, **p < 0.01, or ***p < 0.001.

We hypothesized that probiotic mixtures, rather than single strains, would increase metabolite production. In addition, to identify the metabolites associated with anti-adhesion, antimicrobial activity against pathogens, and anti-inflammatory activity, we evaluated the concentration of SCFAs in the CFSs of nine single strains used in probiotic mixtures and two probiotic mixtures (Consti-Biome and Sensi-Biome) using gas chromatography (Table 2). This study focused on acetic, propionic, and butyric acid production which are predominant SCFAs in the gut [9]. To obtain the CFSs, nine single strains and two probiotic mixtures adjusted to 10^8^ CFU/ml were cultured in MRS broth at 37°C for 24 h. Two probiotic mixtures produced SCFAs, acetic, propionic, and butyric acid, in the range of 5.2–1,489.2 μg/ml. All single strains and probiotic mixtures showed the highest acetic acid concentration among SCFAs ranging from 162.1 to 1,489.2 μg/ml in common. In case of the nine single strains, the *Bifidobacterium* species showed lower acetic acid production (162.1–621.1 μg/ml) than that by other species (937.1–1,181.4 μg/ml). Moreover, propionic and butyric acids exhibited similar concentrations. The concentrations of propionic acids using the single strains were in the range of 4.8–17.1 μg/ml, and butyric acid showed the least concentration among SCFAs in the range of 0.3–1.7 μg/ml. However, butyric acid of *Bifidobacterium bifidum* was not detected. The nine single strains showed total SCFA concentrations of 166.9–1,198.5 μg/ml. The probiotic mixture, Consti-Biome, showed the highest overall SCFA production among all the samples with 1,509.3 μg/ml total SCFA (1,489.2 μg/ml acetic acid, 14.9 μg/ml propionic acid, and 5.2 μg/ml butyric acid), followed by Sensi-Biome with total SCFA concentration of 1,466.1 μg/ml (1,440.2 μg/ml acetic acid, 19.7 μg/ml propionic acid, and 6.2 μg/ml butyric acid) (Fig. 7). These results suggest that our novel probiotic mixtures, Consti-Biome and Sensi-Biome, produced more SCFAs than those by single strains. Therefore, these results explain that the inhibitory abilities of Consti-Biome and Sensi-Biome against pathogens and pro-inflammatory cytokines might be attributed to the SCFAs produced by them.

### Inhibition of Gas Production

**Fig. 8:**
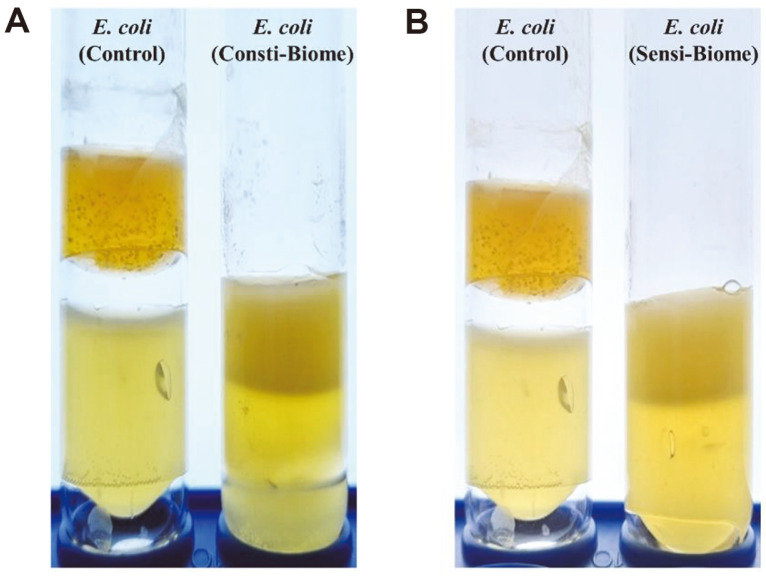
Inhibition of gas production. The lower layer corresponds to the LB agar inoculated with *Escherichia coli* ATCC 8739 and the upper layer is MRS medium with 0.7% agar inoculated with Consti-Biome and Sensi-Biome. In the control tube, the upper layer is MRS medium with 0.7% agar without Consti-Biome and Sensi-Biome. (**A**) Gas production by *E. coli* in LB agar medium as the control (Left) and the inhibitory activity of Consti-Biome (Right). (**B**) Inhibitory activity of Sensi-Biome on gas production by *E. coli*. (Right).

To assess whether Consti-Biome and Sensi-Biome alleviate abdominal bloating caused by pathogens, the inhibition of gas production was evaluated using a nutrient agar medium. *E. coli* was selected owing to its inherent ability to produce a large amount of hydrogen gas resulting from its fermentative activity [30]. In the control tube, gas production was observed when only *E. coli* was cultured in nutrient agar medium. However, gas production was inhibited when *E. coli* was cultured with Consti-Biome or Sensi-Biome (Fig. 8). Therefore, we speculate that Consti-Biome and Sensi-Biome could inhibit gas production by gas-producing pathogens in the host intestine.

## Discussion

Several microorganisms present in the gut are related to host health and the development of some disorders [31]. Imbalance in the gut microbiome, called dysbiosis, leads to recolonization by pathogenic microorganisms, which causes an inflammatory process and has a great influence on the development of a wide range of disorders such as chronic gastrointestinal disorders [32]. Use of probiotics are one of the most promising treatments for various disorders caused by these dysbiosis. Major probiotic bacteria are lactic acid bacteria group including *Lactobacillus*, *Bifidobacterium*, and *Streptococcus* species, and they are known to inhibit the growth of harmful bacteria [33-35]. Previous studies have demonstrated that probiotic bacteria can make metabolites such as SCFA and lactic acid and inhibit the growth of pathogens [9, 29]. In addition, studies have confirmed that inflammatory disorders that can occur in the intestine can be suppressed through immunomodulatory activities [36, 37].

This study aimed to confirm the possibility that newly designed probiotic mixtures, Consti-Biome and Sensi-Biome, can be developed as dietary supplements to inhibit intestinal pathogens through in vitro evaluation. The enteric pathogens used in this study, *S. aureus* and *E. coli*, are representative gram-positive and gram-negative bacteria, respectively, and are potential culprits of intestinal disorders. Previous studies have demonstrated that *S. aureus* [38] and *E. coli* [39, 40] have been linked to and play a causative role in intestinal disorders. Therefore, these two pathogenic strains were selected to evaluate the inhibitory effects of the two probiotic mixtures.

Previous studies have shown the effects of the strains included in Consti-Biome and Sensi-Biome through in vitro studies and clinical trials. Each strain of SynBalance SmilinGut (*B. lactis*, *L. plantarum*, and *L. rhamnosus*), which are present in Consti-Biome, show time-dependent antimicrobial profiles against *S. aureus* and *E. coli*. Moreover, they induce higher secretion levels of the anti-inflammatory cytokine, IL-4, thus lowering TNF-α production than those with sodium dodecyl sulphate treated in murine fibroblast BALB/c 3T3a cell [41]. An clinical study has shown that the administration of these strains alleviates IBS symptoms such as bloating, abdominal pain, constipation, abdominal cramps, and flatulence [14]. In addition, *B. lactis* UABla-12 and *L. acidophilus* DDS-1, which are present in Sensi-Biome, suppress inflammation in LPS-stimulated HT-29 cells [42]. Clinical efficacy of the strains demonstrated that they decrease symptom severity scores related to abdominal pain, distension, bowel habits, and quality of life [15]. These studies predict the effects of Consti-Biome and Sensi-Biome on in vitro mechanisms.

Consistent with the studies, our study demonstrated that each probiotic mixture containing these strains exhibited antimicrobial activity against the two pathogens. However, Consti-Biome and Sensi-Biome were mixed with six probiotic bacteria each containing the strains described above. While this property may be desirable as long as the antimicrobial spectrum of individual strains is limited to pathogenic microbes, it cannot be ruled out that it may affect the normal gut microbiome or other LAB as well [43]. Each single strain used in the probiotic mixtures was able to adhere to the HT-29 cell line in the range of 82–89% (data not shown). However, Consti-Biome and Sensi-Biome, which were mixtures, showed a higher adhesion ratio to HT-29 cells than those by single strains (Fig. 1A). Simultaneously, Consti-Biome and Sensi-Biome inhibited the adhesion of pathogens to HT-29 cells (Fig. 2). These observations suggest that the individual strains contained in Consti-Biome and Sensi-Biome have synergistic effects without negatively affecting each other.

Gut inflammation induced by pathogens alters the microbiota composition and further promotes pathogen growth [8]. Pathogens, toxins, and allergens, such as LPS, cause hypersensitivity by activating antigen-presenting cells [44, 45]. Intestinal bacteria can stimulate or suppress innate immune responses by modulating pro-inflammatory cytokines [46]. Among pro-inflammatory cytokines, TNF-a promotes the secretion of TNF and upregulates the expression of other pro-inflammatory cytokines, such as IL-6 and IL-1β, through nuclear factor-κB activation [6]. Previous studies have demonstrated immunomodulation effects of probiotics. For example, *Lactobacillus* and *Bifidobacterium* species regulate LPS-induced inflammation and downregulate the secretion of inflammatory cytokines in activated macrophages [6, 8, 21]. Similar results were observed in our study, wherein Consti-Biome and Sensi-Biome significantly suppressed the production of TNF-α, IL-6, and IL-1β in LPS-stimulated RAW264.7 cells (Fig. 3), demonstrating potential anti-inflammatory activity.

In order to evaluate the antimicrobial capacity of the Consti-Biome and Sensi-Biome, four protocols were applied as follows; anti-adhesion activity on HT-29 cells, inhibition of growth and biofilm formation, and inhibition of *E. coli* gas production. These four different approaches were designed to mimic acute or chronic infections and to verify the effectiveness of tested probiotics in such conditions. Experimental results demonstrated that the two probiotic mixtures were able to inhibit both pathogens in common under all conditions. Interestingly, the result of competition-based adhesion showed that Consti-Biome had an enhanced inhibitory effect against *S. aureus* while Sensi-Biome had a higher inhibitory effect against *E. coli* (Fig. 2). These results were also confirmed by the experiments of the growth and biofilm formation inhibition tests using CFSs of Consti-Biome and Sensi-Biome (Figs. 4 and 5). Our findings suggest that the Consti-Biome and Sensi-Biome can be used to selectively alleviate disorders caused by *S. aureus* or *E. coli*.

In this study, SEM analysis revealed the CFSs of Consti-Biome and Sensi-Biome caused structural disruptions in *S. aureus* and *E. coli*, respectively (Fig. 6). Damaged bacterial cell membranes lead to increased permeability, leakage of intracellular nucleic acids and proteins, depolarization of membrane potential, and production and accumulation of considerable amounts of reactive oxygen species, resulting in bacterial DNA damage and ultimately bacterial death [47]. Therefore, these results suggest that the antimicrobial substances produced by Consti-Biome and Sensi-Biome can damage the cell membranes of pathogens and cause intracellular substances to leak, eventually causing cell death [20, 47].

We considered the possibility that Consti-Biome and Sensi-Biome could produce specific metabolites with immunomodulatory activity and inhibit the growth of pathogenic strains. In general, LAB inhibit the viability of target microorganisms by producing one or more antimicrobial metabolites, such as organic acids (SCFAs and lactic acid), low molecular weight compounds, antifungal peptides, and antimicrobial peptides (bacteriocins) [24, 48]. The antagonistic activity against pathogens is due to the SCFAs present in the culture supernatant of probiotics [9]. These SCFAs also have an immunomodulatory potential, which implies that they influence the maintenance of anti-inflammatory balance [49].

In this study, the culture supernatants of nine single strains and two probiotic mixtures (Consti-Biome and Sensi-Biome) were analyzed using gas chromatography to compare their abilities to produce SCFAs; it was observed that they can produce acetic, propionic, and butyric acid. (Fig. 7, Table 2.) All the samples, including single strains and probiotic mixtures, produced SCFAs at different levels; however, the concentrations of total SCFAs in two probiotic mixtures were higher than those in single strains. These results support that the two probiotic mixtures can produce more SCFAs than those produced by the single strains through the synergistic effects. SCFAs suppress *S. aureus* invasion into bovine mammary epithelial cells, inhibit *S. aureus*-induced infections [50, 51], and inhibit the growth and virulence of *E. coli* [52]. These data suggest a potential role of SCFAs as immunomodulatory metabolites against pathogen infections. Although these results consider the potential of other bioactive metabolic products, our observations support the hypothesis that SCFAs may, at least partly, explain how Consti-Biome and Sensi-Biome exert anti-adhesion effects, antimicrobial activity against *S. aureus* and *E. coli*, and anti-inflammatory effects.

In addition, several patients with intestinal disorder exhibit abdominal bloating. Several factors contribute to the occurrence of bloating in these patients, and a probable reason could be the production of intestinal gas by intestinal bacteria including some pathogens. Small intestinal bacterial overgrowth, a condition in which microorganisms that should proliferate in the large intestine proliferate excessively in the small intestine, generates a considerable amount of methane or hydrogen gas in the intestine, which stimulates the abdominal wall, causing abdominal pain, bloating, diarrhea, or constipation [53,54]. Additional studies are needed to determine if other gas-producing bacteria can be inhibited. However, our study suggests that gas-induced abdominal bloating caused by gas-producing harmful bacteria is reduced by the administration of Consti-Biome and Sensi-Biome. (Fig. 8)

In conclusion, newly designed two probiotic mixtures, named Consti-Biome and Sensi-Biome, showed (i) inhibitory efficacy against two enteric pathogens, *S. aureus* and *E. coli*, (ii) immunomodulatory effects, and (iii) production of functional metabolites such as SCFAs. Our findings suggest the possibility that the two probiotic mixtures could be developed as dietary supplements to inhibit *S. aureus* and *E. coli*, that may cause intestinal disorders. However, the ability to inhibit other enteric pathogens that can cause intestinal disorders has not been confirmed, and it is necessary to verify whether they have inhibitory effects against intestinal pathogens when actually ingested them. Therefore, further efficacy studies, *in vivo* and clinical trials, are needed to confirm the effectiveness of these probiotic mixtures.

## Supplemental Materials

Supplementary data for this paper are available on-line only at http://jmb.or.kr.

## Figures and Tables

**Table 1:** List of lactic acid bacteria used in the evaluated probiotic mixtures.

	Strain	Origin	Source
Consti-Biome	*Bifidobacterium animalis* ssp. *lactis* BL050 (SynBalance SmilinGut)	Human	Roelmi HPC
	*Lactiplantibacillus plantarum* PBS067 (SynBalance SmilinGut)	Human	Roelmi HPC
	*Lacticaseibacillus rhamnosus* LRH020 (SynBalance SmilinGut)	Human	Roelmi HPC
	*Lactobacillus acidophilus* DDS-1	Human	Chr. Hansen
	*Lactiplantibacillus plantarum* UALp-05	Plant	Chr. Hansen
	*Streptococcus thermophilus* CKDB027	Dairy Food	Chong Kun Dang Bio
Sensi-Biome	*Bifidobacterium bifidum* BB-06	Human	Danisco
	*Bifidobacterium animalis* ssp. *lactis* UABla-12	Human	Chr. Hansen
	*Lactobacillus acidophilus* DDS-1	Human	Chr. Hansen
	*Lactiplantibacillus plantarum* UALp-05	Plant	Chr. Hansen
	*Lactococcus lactis* MG5125	Dairy Food	Mediogen
	*Streptococcus thermophilus* CKDB027	Dairy Food	Chong Kun Dang Bio

**Table 2:** Short-chain fatty acids (SCFAs) production by each single strain and two probiotic mixtures.

	Strain name	Short-chain fatty acids (μg/ml)			
		Acetic acid	Propionic acid	Butyric acid	Total SCFAs
Single strain	*B. bifidum* BB-06	162.1 ± 55.4	4.8 ± 4.2	0.0 ± 0.0	166.9 ± 56.3
	*B. lactis* UABla-12	621.1 ± 459.0	11.4 ± 0.7	0.3 ± 0.6	632.8 ± 458.7
	*B. lactis* BL050	416.0 ± 139.5	13.6 ± 5.2	0.7 ± 0.6	430.3 ± 136.9
	*L. plantarum* PBS067	1,131.8 ± 33.8	13.6 ± 3.7	1.7 ± 0.1	1,147.1 ± 37.2
	*L. rhamnosus* LRH020	1,069.3 ± 232.9	13.0 ± 3.3	1.0 ± 0.2	1,083.2 ± 229.8
	*L. acidophilus* DDS-1	937.1 ± 192.5	17.1 ± 1.9	1.0 ± 0.2	955.2 ± 190.5
	*L. plantarum* UALp-05	1,181.4 ± 130.4	15.5 ± 2.0	1.6 ± 0.3	1,198.5 ± 128.3
	*Lc. lactis* MG5125	1,158.9 ± 30.1	7.4 ± 0.1	1.4 ± 0.4	1,167.7 ± 30.2
	*S. thermophilus* CKDB027	1,012.0 ± 33.0	8.9 ± 4.2	1.2 ± 0.3	1,022.0 ± 30.4
Probiotic mixtures	Consti-Biome	1,489.2 ± 59.3	14.9 ± 11.6	5.2 ± 5.5	1,509.3 ± 60.9
	Sensi-Biome	1,440.2 ± 119.3	19.7 ± 20.9	6.2 ± 3.7	1,466.1 ± 95.8

All values are mean ± standard deviation.
