# Supplementary material for: In Vitro Evaluation of Probiotic Properties of Two Novel Probiotic Mixtures, Consti-Biome and Sensi-Biome
Source: J Microbiol Biotechnol. 2023 Jun 19;33(9):1149–61. doi: 10.4014/jmb.2303.03011 (PMC10580887; doi:10.4014/jmb.2303.03011)
Supplement: Supplementary file 1 [file jmb-33-9-1149-supple.pdf]

J. Microbiol. Biotechnol.
https://doi.org/10.4014/jmb.2303.03011

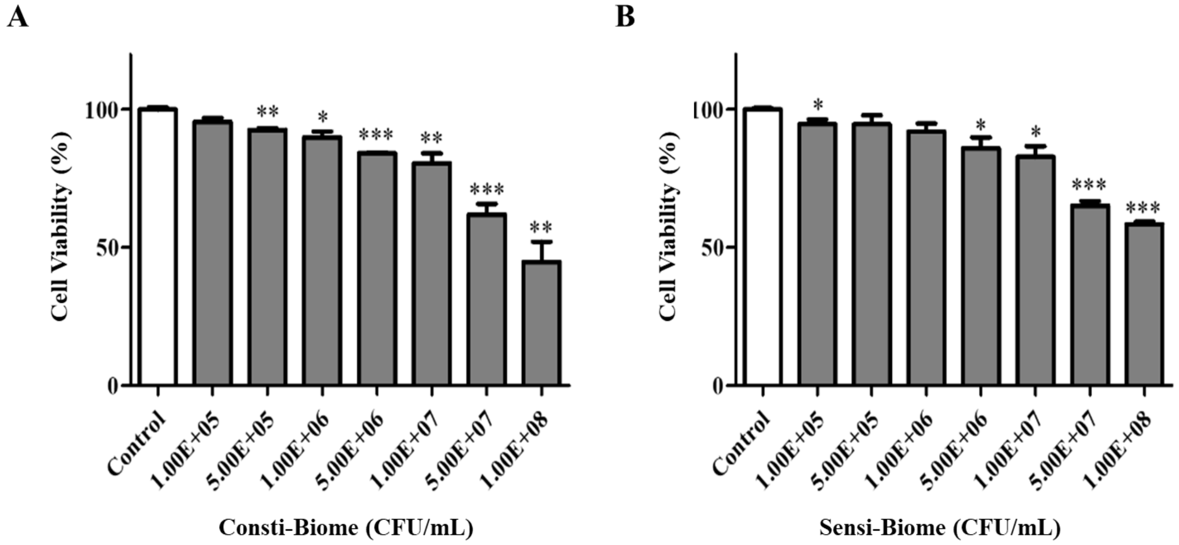

Supplementary Fig. S1. Effect of Consti-Biome and Sensi-Biome on RAW264.7 cell viability.

RAW264.7 cells were seeded in 96-well plates for 24 h. Cells were treated with (A) Consti-Biome or (B) Sensi-Biome in a dose-dependent manner for 24 h. Viability was assessed by MTT assay and expressed as a percentage (%) of control. The values are expressed as the mean ± SD. A significant difference from the control was indicated as, * p <0.05, ** p <0.01, or *** p <0.001.
